# Different Neural Systems Contribute to Semantic Bias and Conflict Detection in the Inclusion Fallacy Task

**DOI:** 10.3389/fnhum.2014.00797

**Published:** 2014-10-20

**Authors:** Peipeng Liang, Vinod Goel, Xiuqin Jia, Kuncheng Li

**Affiliations:** ^1^Xuanwu Hospital, Capital Medical University, Beijing, China; ^2^Brain Key Laboratory of Magnetic Resonance Imaging and Brain Informatics, Beijing, China; ^3^Department of Psychology, York University, Toronto, ON, Canada; ^4^IRCCS Fondazione Ospedale San Camillo, Venice, Italy

**Keywords:** fMRI, inductive reasoning, prefrontal cortex, inclusion fallacy, category-based induction

## Abstract

The inclusion fallacy is a phenomenon in which generalization from a specific premise category to a more general conclusion category is considered stronger than a generalization to a specific conclusion category nested within the more general set. Such inferences violate rational norms and are part of the reasoning fallacy literature that provides interesting tasks to explore cognitive and neural basis of reasoning. To explore the functional neuroanatomy of the inclusion fallacy, we used a 2 × 2 factorial design, with factors for quantification (explicit and implicit) and response (fallacious and non-fallacious). It was found that a left fronto-temporal system, along with a superior medial frontal system, was specifically activated in response to fallacious responses consistent with a semantic biasing of judgment explanation. A right fronto-parietal system was specifically recruited in response to detecting conflict associated with the heightened fallacy condition. These results are largely consistent with previous studies of reasoning fallacy and support a multiple systems model of reasoning.

## Introduction

As rational beings, we look to reasons to motivate and justify our actions. However, a long series of cognitive studies suggest that we make systematic errors while reasoning. Perhaps the most pervasive errors have to do with the impact of our belief structures on logical reasoning (Wilkins, 1928; Evans et al., 1983). Several imaging studies have examined the neural basis of belief bias (i.e., the inclination to agree or disagree with an argument based upon whether we find the conclusion believable or unbelievable) in syllogistic reasoning (Goel et al., 2000; Goel and Dolan, 2003). The basic finding is that the left frontal–temporal system is recruited for logical reasoning in the presence of semantic content about which subjects have beliefs, and a right frontal and bilateral parietal system is engaged where such beliefs are absent (Goel et al., 2000) or need to be overcome to generate the logical response (Goel and Dolan, 2003). Where the beliefs are not overcome, a ventral medial frontal system is engaged (Goel and Dolan, 2003). The goal of the current study is to see if these mechanisms generalize to more informal reasoning domains, such as category-based induction.

Category-based induction is a reasoning process by which we project knowledge about certain classes of entities to other related classes of entities (e.g., inferring that ostriches have gene X from the fact that robins have gene X). Inductive generalization from the known to the unknown enables us to benefit from past instances and enlarge the scope of our knowledge. There is a phenomenon within this domain, known as the inclusion fallacy. The inclusion fallacy is a phenomenon in which generalization from a specific category to a more general category (e.g., from robin to bird) is considered to be stronger or more convincing than generalization to a more specific category (e.g., to ostrich) nested within the more general set. Consider the following examples from Osherson et al. (1990):

Robins secrete uric acid crystals

– – – – – – – – – – – – – – – – –

Birds secrete uric acid crystals

and

Robins secrete uric acid crystals

– – – – – – – – – – – – – – – – – – –

Ostriches secrete uric acid crystals.

Subjects are presented with pairs of arguments, such as these, and required to make a direct comparison of their relative strength. Many (but not all) people sometimes (but not always) fallaciously choose the first argument as stronger than the second, and thus, commit the inclusion fallacy (since the conclusion of the second argument is contained in the conclusion of the first, it can not be stronger).

The individual arguments are inductive and have no logically correct response. However, as typically administered (Osherson et al., 1990), the task forces subjects to make a direct comparison of the relative strength of the two arguments. There is a logically correct response to this critical component of the task. It is to say that the generalization to all birds cannot be stronger than the generalization to a specific bird (and vice versa). This response is, however, excluded by the task setup. Subjects must choose one or the other as being “stronger,” there being no option to say “same strength”^1^. None the less, it seems to defy rational plausibility norms to assert a property to all birds but not a specific bird.

The inclusion fallacy seems to reflect the perceived relationship between the subjects in the premise and conclusion. The link between robin and bird is quite strong because robin is considered to be a typical/central member of the bird category. But an ostrich, despite being a bird, is an atypical/peripheral member of the bird category and is somewhat removed from the representation of robin. In this sense, the phenomenon of inclusion fallacy is similar to the conjunction fallacy in the Linda problem^2^ (Tversky and Kahneman, 1983) and the belief-bias effect in deductive reasoning (Evans et al., 1983; Goel and Dolan, 2003; Evans and Curtis-Holmes, 2005; De Neys, 2006a,b), in that the fallacious response is biased by the organization of our world knowledge. However, participants will sometimes overlook the more constrained/logical response and answer on the basis of their knowledge about birds, robins, and ostriches. The inference is biased toward the more familiar/easily accessible category (bird over ostrich).

Not all participants are susceptible to the inclusion fallacy, and those that are do not fall prey to it on all occasions. One factor that may affect participants’ susceptibility to the fallacy is the quantifier associated with the conclusion. In the stimuli used by Osherson et al. (1990), e.g., “birds secrete uric acid crystals,” the quantifier is only implied, leaving room for ambiguity. If one assumes a strict universal quantifier (e.g., “all birds secrete uric acid crystals”) then one should be more aware of the fact that the superordinate category (i.e., bird) subsumes the subordinate category (i.e., ostrich), which should in turn reduce the inclusion fallacy. However, if one does not assume strict universal quantification, then one may be less likely to subsume the subordinate category in the superordinate category. For example, the participant may reason that perhaps the sentence means “most birds or virtually all birds. And after all, ostriches are not real birds.” Under such an interpretation one is more likely to make the inclusion fallacy. Thus, the absence of an explicit “all” should increase uncertainty and the inclusion fallacy while the presence of an explicit “all” should decrease uncertainty and the fallacy response. That the presence of an explicit or implicit quantifier should modulate the inclusion fallacy is consistent with the psychological literature on the interpretation of quantifiers (Collins and Quillian, 1969; Newstead and Griggs, 1984). It is also consistent with a related study (Sloman, 1998) that shows that fallacious inferences (specifically, the inclusion similarity)^3^ can be modulated by making the category of inclusion relations explicit.

To understand the neural basis of the inclusion fallacy, and its modulation by explicit and implicit quantifiers, we undertook an fMRI study of healthy volunteers while they engaged in generalization inferences on material similar to Osherson et al. (1990). At the behavioral level, we anticipated that a subset of the participants would display the inclusion fallacy and that the fallacy would be displayed much more frequently in the implicit quantifier condition than the explicit quantifier condition. At the neural level, we were interested in the mechanisms underlying responses biased by beliefs and knowledge structures (i.e., the fallacious responses) versus responses in which these beliefs and knowledge structures were bypassed/suppressed to generate non-fallacious responses. We expected these systems to be modulated by the explicit/implicit quantifier condition. Based on the fact that fallacious responses are driven by the organization of our beliefs, we predicted involvement of a left hemisphere frontal–temporal system, including left middle/inferior frontal gyrus and middle temporal gyrus in this condition as seen in several previous reasoning studies (Goel et al., 2000; Goel and Dolan, 2004). Reasoning trials uninfluenced by beliefs (i.e., the non-fallacious responses in the present study), on the other hand, should activate a parietal system, often found in reasoning trials devoid of beliefs (Goel et al., 2000; Waechter et al., 2012). The task paradigm contains a tension/conflict between the fallacious and non-fallacious responses. This is exasperated in the implicit quantifier condition where the uncertain scope of the quantifier leaves room for doubt (see Discussion). In this situation, we predicted activation in right frontal PFC in response to conflict detection, particularly in the case of non-fallacy responses (Goel et al., 2000; Goel and Dolan, 2003; De Neys et al., 2008; Stollstorff et al., 2011).

## Materials and Methods

### Subjects

Sixty-two paid healthy undergraduate and postgraduate students participated in the experiment. All subjects were right-handed and had normal or corrected-to-normal vision. None of the subjects reported any history of neurological or psychiatric diseases. The study was approved by the Ethics Committee of Xuanwu Hospital, Capital Medical University. All participants gave written informed consent.

### Stimuli and design

One hundred twenty trials, modeled on the Osherson et al. (1990) stimuli, were included in the current study. Each trial was composed of pairs of arguments, one appearing above the other (see Table 1). The ordering of the arguments was counterbalanced. The subjects were instructed to judge, and indicate, which one of the two arguments was stronger.

**Table 1:** **Example of experimental tasks**.

	Explicit	Implicit
**Argument 1** (typical to atypical)	All robins secrete uric acid crystals	Robins secrete uric acid crystals
	All ostriches secrete uric acid crystals	Ostriches secrete uric acid crystals
**Argument 2** (typical to general)	All robins secrete uric acid crystals	Robins secrete uric acid crystals
	All birds secrete uric acid crystals	Birds secrete uric acid crystals

The stimuli were divided into two conditions (see Table 1), explicit quantification (60), and implicit quantification (60). Subjects’ responses to each trial were used to further divide the stimuli into fallacy or non-fallacy response trials. A fallacious response would be one where the participant chose the argument “robins secrete uric acid crystals, therefore, birds secrete uric acid crystals” as being stronger or more convincing than “robins secrete uric acid crystals, therefore, ostriches secrete uric acid crystals.” The reverse selection (i.e., where the latter is stronger or more convincing than the former) would be the non-fallacious correct selection. This yielded a 2 × 2 factorial design, with factors for quantification (explicit and implicit) and response (fallacious or non-fallacious), resulting in the following four cells: implicit fallacy (I_F), implicit non-fallacy (I_NF), explicit fallacy (E_F), and explicit non-fallacy (E_NF).

### Stimuli presentation

Stimuli from all conditions were organized into two sessions and presented randomly in an event related design. The order of sessions was counterbalanced among subjects. Trials began with the presentation of one of the arguments (premise plus conclusion). Two seconds later, the second argument (premise plus conclusion) was presented and subjects were given 8 s to respond. Half of the participants used a left button press to indicate that the first argument was stronger and the right button press to indicate that the second argument was stronger. The other half of the participants used the reverse. The two arguments remained on the screen until the end of the trial or the subjects’ button-press response. Subjects were instructed to respond as accurately and quickly as possible and move to the next trial if the stimuli advanced before they could respond. The length of trials varied from 9 to 11 s (with a TR/2 jitter), i.e., the length of the trials may be 9, 10, or 11 s with the same probability, randomly. This was determined by pilot data indicating that the range of the inter-trial interval was 7–9 s, with a reaction time of around 3 s. There were 60 event presentations during a session and each session lasted 10 min.

### MRI data acquisition

Scanning was performed on a 3.0-T MRI system (Siemens Trio Tim; Siemens Medical System, Erlanger, Germany) and with a 12-channel phased array head coil. Foam padding and headphones were used to limit head motion and reduce scanning noise. High-resolution structural images were acquired using a T1 weighted 3D MPRAGE sequence (TR/TE = 1600/2.25 ms, TI = 800 ms, 192 sagittal slices, FOV = 256 mm, 9° flip angle, voxel size = 1 mm × 1 mm × 1 mm). Functional images were obtained using a T2* gradient-echo EPI sequence (TR/TE = 2000/31 ms, 90° flip angle, 64 × 64 matrix size in 240 mm × 240 mm FOV). Thirty axial slices with a thickness of 4 mm and an interslice gap of 0.8 mm were acquired and paralleled to the AC–PC line. The scanner was synchronized with the presentation of every trial.

### Data preprocessing

Data were analyzed using SPM5 software ^4^. The first four images for each session were discarded to allow for T1 equilibration effects. The remaining fMRI images were first corrected for within-scan acquisition time differences between slices and then realigned to the first volume to correct for inter-scan head motions (head movements were <1 voxel in all cases). The structural image was co-registered to the mean functional image created from the realigned images using a linear transformation. The transformed structural images were then segmented into gray matter (GM), white matter (WM), and cerebrospinal fluid (CSF) by using a unified segmentation algorithm (Ashburner and Friston, 2005). The realigned functional volumes were spatially normalized to the Montreal Neurological Institute (MNI) space and re-sampled to 3 mm isotropic voxels using the normalization parameters estimated during unified segmentation. The registration of the functional data to the template was checked for each individual subject. Subsequently, the functional images were spatially smoothed with a Gaussian kernel of 8 mm × 8 mm × 8 mm full width at half maximum (FWHM) to decrease spatial noise.

### fMRI analysis

For all trials, the epoch of interest extends from the presentation of the first argument to the response. The BOLD signal was modeled using canonical HRF with temporal derivative implemented in SPM5. Condition effects at each voxel were estimated according to the general linear model and regionally specific effects were compared using linear contrasts. Each contrast produced a statistical parametric map (SPM) of the *t*-statistic, which was subsequently transformed to a unit normal *Z*-distribution. The contrast images were then used in a random effect analysis to determine the regions most consistently activated across subjects. The contrasts of primary interest in the present study are the main effect of fallacy (F–NF, NF–F), explicitness (I–E and E–I), and the interaction effects [(I_F–I_NF)–(E_F–E_NF) and (E_F–E_NF)–(I_F–I_NF)]. The activations reported survived a voxel-level threshold of *p* < 0.001 and a cluster size comprised of a minimum of eight contiguous voxels, which corresponded to a corrected *p* < 0.05 using the AlphaSim program ^5^ (parameters: FWHMx = 12.23 mm, FWHMy = 10.39 mm, FWHMz = 9.67 mm, within the GM mask). The real smoothness in the three directions was estimated by using 3dFWHMx.

## Results

### Behavioral performance

Of the 62 subjects, 58 exhibited the fallacy at least once in the implicit condition and 54 exhibited the fallacy at least once in the explicit condition. To ensure adequate signal-to-noise ratio, and to allow for within subject analyses, we used a cut off of at least 12 trials in the fallacy and logical response conditions to select participants for fMRI analyses. Fifteen subjects (7 females) with a mean age of 23.6 ± 3.1 years met this criterion and were included in the subsequent fMRI data analysis. The initial behavioral analysis, below, includes all 62 participants. The subsequent analysis is limited to 15 participants used in the fMRI analysis. The pattern of results in the two cases is identical.

**Figure 1:**
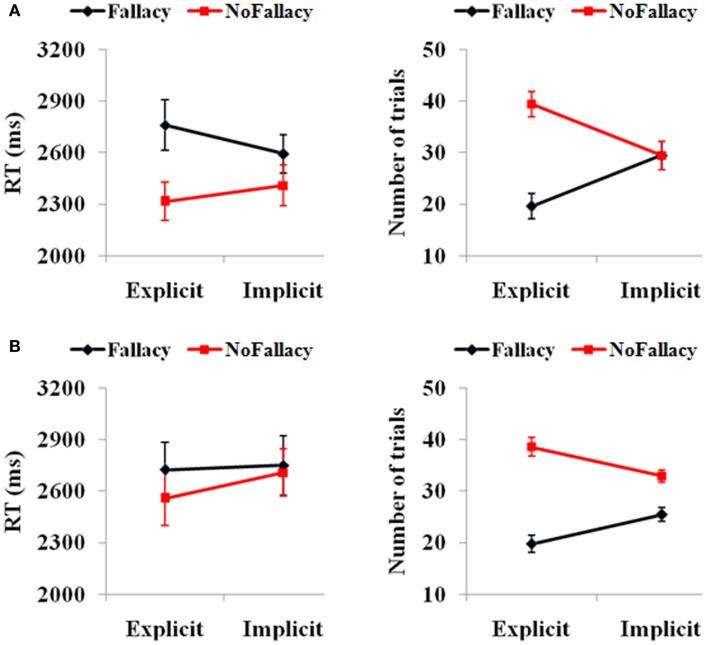
**(A)** Behavioral performance of 62 subjects and **(B)** the 15 subjects with enough trials for the further fMRI data analysis. The error bars represent the SEM.

Behavioral scores were in keeping with expectations (see Figure 1). In terms of responses from all 62 participants, we found a main effect of response [*F*(1,61) = 3.81, *p* = 0.05], such that the number of non-fallacious responses were greater than the number of fallacious responses. There was also a quantification (explicit, implicit) by response (fallacy, non-fallacy) interaction [*F*(1,61) = 23.97, *p* = 0.00] (see Figure 1A), driven by the fact that there were more non-fallacious responses than fallacious responses in the explicit quantifier trials [*F*(1,61) = 15.54, *p* = 0.00], but there was no difference in the number of non-fallacious and fallacious responses in the implicit trials [*F*(1,61) = 0.02, *p* = 0.90].

In terms of reaction times, there was a main effect of response [*F*(1,49) = 6.15, *p* = 0.017], with participants taking longer to respond in trials in which they commit the inclusion fallacy (see Figure 1A). The main effect of quantification [*F*(1,49) = 0.24, *p* = 0.62] and the quantification by response interaction [*F*(1,49) = 2.68, *p* = 0.11] were not significant. The *post hoc* analysis of RTs also showed that the RT for fallacy trials was significantly longer than that for non-fallacy response trials in the explicit condition [*F*(1,52) = 4.20, *p* = 0.046] but not in the implicit condition [*F*(1,55) = 2.28, *p* = 0.14]. (Note: as there are NULL values for RT in some conditions for several subjects, the degrees of freedom are not always 61, but variable).

We then analyzed the results of the 15 subjects that will be included in the fMRI analyses (see Figure 1B). In terms of accuracy responses, we found a main effect of response [*F*(1,14) = 24.47, *p* = 0.00], such that the number of non-fallacious responses was greater than the number of fallacious responses, and a quantification (explicit, implicit) by response (fallacy, non-fallacy) interaction [*F*(1,14) = 11.70, *p* = 0.004], again driven by the fact that the difference between non-fallacious and fallacious responses was greater in the explicit trials than the implicit trials. In terms of reaction times, the effects were not significant, but the pattern was similar to that of the 62 subjects.

### fMRI results

As noted above, the fMRI results are based on 15 of the 62 participants who had at least 12 trials in each of the 4 conditions.

**Figure 2:**
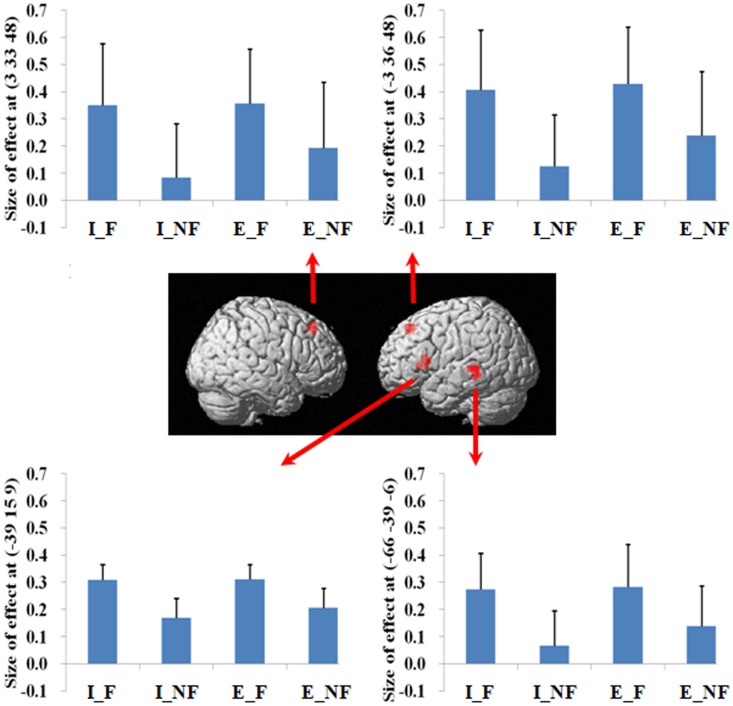
**A statistical parametric map (SPM) rendered into standard stereotactic space**. A comparison of fallacy trials versus non-fallacy trials (F_NF) results in activation in left inferior frontal gyrus/insula (MNI: −39, 15, 9; *T* = 4.81) (BA 45/13), left middle temporal gyrus (MNI: −66, −39, −6; *T* = 5.21) (BA 21/22), left medial frontal gyrus (MNI: −3, 36, 48; *T* = 4.60) (BA 8), and right superior frontal gyrus (MNI: 3, 33, 48; *T* = 5.23) (BA 8) [also see the main effect of (F–NF) in Table 2]. Condition specific parameter (beta) estimates show that the left fronto-temporal system and bilateral mesial frontal gyrus are specifically responding to fallacy trials in both implicit and explicit conditions. The error bars represent the SEM. The activations reported survived an uncorrected voxel-level intensity threshold of *p* < 0.001 with a minimum cluster size of 10 contiguous voxels, which corresponds to a corrected *p* < 0.05 (using the AlphaSim program as described in Section Materials and Methods).

The main effect of response (Table 2), derived from comparisons of trials with fallacious and non-fallacious responses (F–NF), revealed activation of bilateral superior/medial frontal gyrus (BA 8), left inferior frontal gyrus/insula (BA45, 13), and left middle temporal gyrus (BA 21, 22) in the fallacy trials (Table 2; Figure 2). The reverse comparison, of the main effect of response, non-fallacious versus fallacious trials (NF–F), revealed no significant activations.

**Table 2:** **Main effect of fallacy and explicitness and the interaction effect of fallacy by explicitness**.

Brain regions	MNI coordinate			BA	Cluster size	*T*-score
	*x*	*y*	*z*	
**F–NF**				
Medial⋅Superior frontal gyrus	3	33	48	8	17	5.23
Lt. middle temporal gyrus	−66	−39	−6	21	28	5.21
Lt. middle temporal gyrus	−63	−36	3	22		4.58
Lt. inferior frontal gyrus/insula	−39	15	9	45/13	17	4.81
	−30	24	6	45		4.64
Lt. medial frontal gyrus	−3	36	48	8	12	4.60
**NF–F**				
No significant activation						
**E–I**				
No significant activation						
**I–E**				
Rt. inferior parietal lobule	42	−54	48	40	10	5.02
Rt. superior parietal lobule	36	−57	54	7		3.87
**(I_F–I_NF)–(E_F–E_NF)**				
Rt. superior parietal lobule	27	−57	45	7	33	4.74
Rt. precuneus	24	−72	51	7		3.37
Lt. fusiform gyrus	−48	−57	−15	37	10	4.22
Rt. middle frontal gyrus	48	33	18	46	10	3.70
**(E_F–E_NF)–(I_F–I_NF)**				
No significant activation						

**Figure 3:**
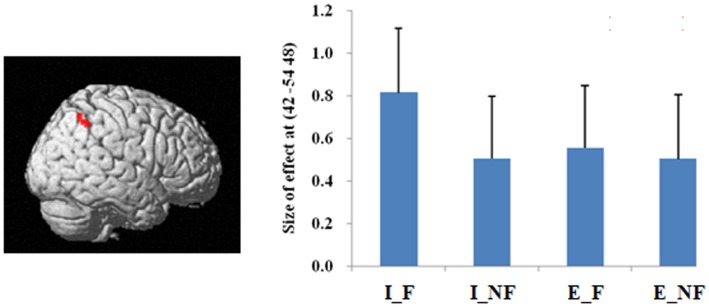
**A statistical parametric map (SPM) rendered into standard stereotactic space**. A comparison of implicit trials versus explicit trials (I–E) results in activation in right inferior/superior parietal lobule (MNI: 42, −54, 48/36, −57, 54; *T* = 5.02/3.87) (BA 40/7) [also see the main effect of (I–E) in Table 2]. Condition specific parameter (beta) estimates show that the right parietal area is responding to fallacy trials in both implicit and explicit conditions, but the main effect in this region is mainly driven by the implicit fallacy trials. The error bars represent the SEM. The activations reported survived an uncorrected voxel-level intensity threshold of *p* < 0.001 with a minimum cluster size of 10 contiguous voxels, which corresponds to a corrected *p* < 0.05 (using the AlphaSim program as described in Section Materials and Methods).

The main effect of quantification, derived from comparisons of implicit minus explicit trials, revealed activation of right superior/inferior parietal lobule (BA 40, 7) (Table 2; Figure 3). The reverse comparison, explicit minus implicit quantifiers, revealed no significant activations.

**Figure 4:**
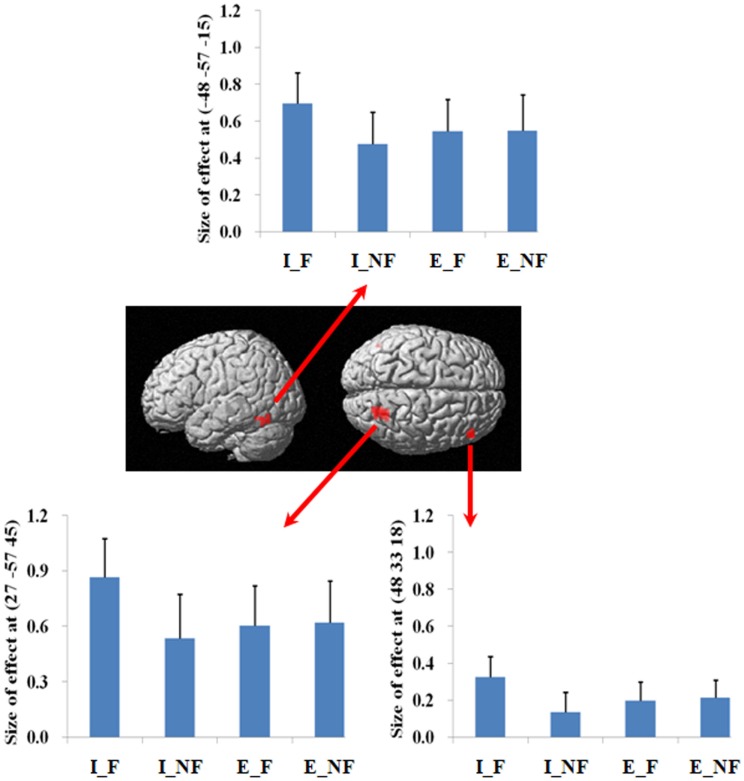
**A statistical parametric map (SPM) rendered into standard stereotactic space**. The quantification (explicit, implicit) by response (fallacious, non-fallacious) interaction, i.e., a comparison of the difference between implicit fallacy trials versus implicit non-fallacy trials with the difference between explicit fallacy trials versus explicit non-fallacy trials [(I_F–I_NF)–(E_F–E_NF)], results in activation in right middle frontal gyrus (MNI: 48, 33, 18; *T* = 3.70) (BA 46) and superior parietal lobule (MNI: 27, −57, 45; *T* = 4.74) (BA 7) [also see the interaction effect of (I_F–I_NF)–(E_F–E_NF) in Table 2]. Condition specific parameter (beta) estimates show that the right fronto-parietal system is specifically responding to fallacies with implicit items, but not to fallacies with explicit items. The error bars represent the SEM. The activations reported survived an uncorrected voxel-level intensity threshold of *p* < 0.01 with a minimum cluster size of 10 contiguous voxels, which corresponds to a corrected *p* < 0.05 (using the AlphaSim program as described in Section Materials and Methods).

We next examined the interaction between response and quantification. The difference between fallacious and non-fallacious responses in implicit condition trials [(I_F–I_NF)–(E_F–E_NF)], resulted in greater activation in right middle frontal gyrus (BA 46), right superior parietal lobule (BA 7), and left fusiform gyrus (BA 37) than the difference between fallacious and non-fallacious responses in the explicit condition trials (Table 2; Figure 4). No regions of significant activation were found in the reverse direction [(I_NF–I_F)–(E_NF–E_F)].

Additionally, in order to exclude the potential effect of task difficulty on the activations, we performed another analysis using RT of each trial as covariates. These results are reported in Table S1 in Supplementary Material. It was found that almost all activations survived the supplementary analysis, indicating that the results were not driven by task difficulty differences between trial types.

## Discussion

Consistent with previous literature (Osherson et al., 1990; Shafir et al., 1990), our results demonstrate susceptibility to the inclusion fallacy in a subset of participants. Furthermore, we demonstrate that the fallacy is indeed modulated by the explicitness of the quantifier. The presence of an explicit universal quantifier significantly reduces the rate of fallacious responses. This may be because the explicit quantifier eliminates ambiguity regarding the scope of the general category and increases the likelihood that the general category will subsume the more specific category.

Our main aim is to explore the neural basis of this fallacy and its modulation by explicit quantification. Consistent with our first neural prediction we found that committing the fallacy was associated with a predominantly left hemisphere frontal–temporal system, including the left inferior frontal gyrus/insula and middle temporal gyrus. This is a semantic system found to be involved in inductive reasoning and belief-based deductive reasoning (Goel et al., 2000; Goel and Dolan, 2004). The involvement of this system in the fallacious response trials is consistent with the possibility that fallacious responses in this paradigm are driven by a combination of the organization of our knowledge base (i.e., typicality/centrality effects), which sometimes exclude ostriches from the class of birds, and an overweighting of the resulting belief-based response over the more rationally plausible response. The activity in bilateral medial/superior frontal cortex may be associated with attentional orientation response (Hopfinger et al., 2000; Rushworth et al., 2004; Woldorff et al., 2004; Taylor et al., 2008).

Despite our prediction of parietal activation, we did not find significant activation in the reverse condition (non-fallacious responses versus fallacious responses). One possible explanation for the lack of finding in this comparison is that, unlike the syllogistic reasoning paradigm, where the logical response is much more complex and effortful, in the present paradigm the non-fallacious response is trivial, so activations associated with it may have been subsumed by the fallacy condition.

In terms of the quantification factor, the absence of the explicit quantifier significantly increased the number of fallacious responses and decreased the number of non-fallacious responses. The neural correlates of this can be seen in the activation of right inferior and superior parietal lobule in the comparison of implicit versus explicit conditions. The implicit condition introduces some uncertainty into the task by increasing ambiguity. Parameter estimates (Figure 4) indicate that this activation is driven by the difference in implicit fallacious versus implicit non-fallacious responses. We consider this activation below, in the discussion of the interaction results.

The explicit minus implicit comparison, on the other hand, revealed no significant activation. As above, it is possible that, given the explicit condition had a preponderance of non-fallacious responses, and that the non-fallacious condition is quite trivial (if the fallacious response is never considered), activations associated with the explicit quantifier condition may be subsumed by activations in the implicit quantifier condition.

Focusing on the response by quantifier interaction highlights the critical role of right lateral prefrontal cortex and parietal lobule system in reasoning. As this is an interaction analysis, and controls for the presence of fallacy and non-fallacy responses, one can interpret the result as being driven by the greater uncertainty in the implicit condition rather than general semantic requirements of the fallacy responses (as in the main effect). (Examination of the parameter estimates clearly indicates that the effect is driven by differential response of this system to the fallacious versus non-fallacious responses in the implicit condition. This right hemisphere frontal parietal system shows no differential sensitivity to the explicit condition trials.) When one exhibits the fallacy in the explicit condition (i.e., after being told that *All* birds have X) it may be a function of oversight, or simply believing that the property of the superordinate category does not generalize to this specific subordinate category (e.g., believing that most properties of robins do not generalize to ostriches). However, the implicit condition facilitates the fallacy by introduction of uncertainty and ambiguity. In the absence of an explicit quantifier, one may be less likely to subsume the subordinate category in the superordinate category. For example, the participant may reason that perhaps the sentence means “most birds or virtually all birds. And after all, ostriches are not real birds.” Under such an ambiguous interpretation, one is more likely to make the inclusion fallacy.

These results differ in two important respects from our expectations. First, the activation was not specific to the non-fallacious condition (i.e., where the fallacious response is suppressed), as we had predicted. Previous studies have reported right PFC activation in detecting and/or overcoming conflict in reasoning (Goel et al., 2000; Goel and Dolan, 2003; Aron et al., 2004; Prado and Noveck, 2007; De Neys et al., 2008; Stollstorff et al., 2011). However, there is evidence that fallacious responses are accompanied by an awareness of the conflict between the more logical response and the belief cued response, even when the fallacious response is not suppressed (De Neys, 2006a,b). The present results suggest that detection of conflict may be sufficient to activate this system. Second, while several previous studies report right PFC activation for conflict detection, Goel and Dolan (2003) also noted accompanying activation in parietal cortex, even though it did not survive correction. The present results suggest a role of the parietal system in conflict detection. Finally, the recruitment of the left fusiform gyrus is consistent with semantic processing and retrieval (Thompson-Schill et al., 1999; Devlin et al., 2006; Mion et al., 2010).

In summary, our results show that a left fronto-temporal system, along with bilateral medial superior frontal system, is specifically activated in the main effect of fallacy in response to biasing of reasoning judgment by the semantic organization of knowledge. A right fronto-parietal system, along with left fusiform gyrus, is specifically recruited in the absence of explicit quantifiers, where fallacious responses increase, as a function of increased uncertainty and ambiguity. These activations may reflect an awareness of the conflict between the selected response and logical response. More generally, these results reinforce the involvement of multiple systems in logical reasoning.

## Conflict of Interest Statement

The authors declare that the research was conducted in the absence of any commercial or financial relationships that could be construed as a potential conflict of interest.

## Supplementary Material

The Supplementary Material for this article can be found online at http://www.frontiersin.org/Journal/10.3389/fnhum.2014.00797/abstract

## Footnotes

^1^It remains an open question whether the fallacious response would persist if a “same strength” option was made available to participants.

^2^Like the inclusion fallacy, the conjunction fallacy requires a contrivance whereby the one piece of information that appears individually and in the conjunct (i.e., Linda is a bank teller) is not in keeping with the description of Linda, whereas the other half of the conjunct is.

^3^Inclusion similarity is the phenomenon whereby the first argument below is considered stronger (or more convincing) than the second argument: (A) all animals use norepinephrine as a neurotransmitter. Therefore, all mammals use norepinephrine as a neurotransmitter. (B) All animals use norepinephrine as a neurotransmitter. Therefore, all reptiles use norepinephrine as a neurotransmitter. The rationale is that the class of mammals is considered to be more representative or similar to the class of animals than is the class of reptiles.

^4^http://www.fil.ion.ucl.ac.uk

^5^http://afni.nimh.nih.gov/pub/dist/doc/manual/AlphaSim.pdf
