# Supplementary material for: Different Neural Systems Contribute to Semantic Bias and Conflict Detection in the Inclusion Fallacy Task
Source: Front Hum Neurosci. 2014 Oct 20;8:797. doi: 10.3389/fnhum.2014.00797 (PMC4202773; doi:10.3389/fnhum.2014.00797)
Supplement: Supplementary file 1 [file Table1.PDF]

All stimuli used in this study. All of them were written in Chinese. Totally, we had 120 trials, with 60 explicit trials and 60 implicit trials.

Part 1 Implicit tasks

No.	Up	Down
1	猫有T细胞，因此，麋鹿有T细胞	猫有T细胞，因此，四足动物有T细胞
2	麻雀有籽骨，因此，火烈鸟有籽骨	麻雀有籽骨，因此，鸟有籽骨
3	蚂蚁含有内质网，因此，蠕虫含有内质网	蚂蚁含有内质网，因此，昆虫含有内质网
4	鲈鱼有一个鳔，因此，甲鱼有一个鳔	鲈鱼有一个鳔，因此，鱼有一个鳔
5	苹果含有维生素D，因此，油桃含有维生素D	苹果含有维生素D，因此，水果含有维生素D
6	胡萝卜含有铁，因此，青椒含有铁	胡萝卜含有铁，因此，蔬菜含有铁
7	松树在钙质土壤生长良好，因此，桃树在钙质土壤生长良好	松树在钙质土壤生长良好，因此，树在钙质土壤生长良好
8	马有alpha属性，因此，四足动物有alpha属性	马有alpha属性，因此，蜥蜴有alpha属性
9	鸽子有beta属性，因此，鸟有beta属性	鸽子有beta属性，因此，模仿鸟有beta属性
10	蜘蛛有肺脏，因此，昆虫有肺脏	蜘蛛有肺脏，因此，螳螂有肺脏
11	金鱼含有巨噬细胞，因此，鱼含有巨噬细胞	金鱼含有巨噬细胞，因此，鲸鱼含有巨噬细胞
12	香蕉含有钾，因此，水果含有钾	香蕉含有钾，因此，甜瓜含有钾
13	番茄含有抗氧化剂，因此，蔬菜含有抗氧化剂	番茄含有抗氧化剂，因此，马铃薯含有抗氧化剂
14	郁金香易得灰霉病，因此，花易得灰霉病	郁金香易得灰霉病，因此，鸢尾花易得灰霉病
15	白杨树有22对染色体，因此，树有22对染色体	白杨树有22对染色体，因此，锡卡莫尔树有22对染色体
16	猫有T细胞，因此，狗有T细胞	猫有T细胞，因此，四足动物有T细胞
17	麻雀有籽骨，因此，鹦鹉有籽骨	麻雀有籽骨，因此，鸟有籽骨
18	蚂蚁含有内质网，因此，苍蝇含有内质网	蚂蚁含有内质网，因此，昆虫含有内质网
19	鲈鱼有一个鳔，因此，金鱼有一个鳔	鲈鱼有一个鳔，因此，鱼有一个鳔
20	苹果含有维生素D，因此，橘子含有维生素D	苹果含有维生素D，因此，水果含有维生素D
21	胡萝卜含有铁，因此，青椒含有铁	胡萝卜含有铁，因此，蔬菜含有铁
22	松树在钙质土壤生长良好，因此，桃树在钙质土壤生长良好	松树在钙质土壤生长良好，因此，树在钙质土壤生长良好
23	马有alpha属性，因此，四足动物有alpha属性	马有alpha属性，因此，狮子有alpha属性
24	鸽子有beta属性，因此，鸟有beta属性	鸽子有beta属性，因此，麻雀有beta属性
25	蜘蛛有肺脏，因此，昆虫有肺脏	蜘蛛有肺脏，因此，蜜蜂有肺脏
26	金鱼含有巨噬细胞，因此，鱼含有巨噬细胞	金鱼含有巨噬细胞，因此，鳟鱼含有巨噬细胞
27	香蕉含有钾，因此，水果含有钾	香蕉含有钾，因此，葡萄含有钾
28	番茄含有抗氧化剂，因此，蔬菜含有抗氧化剂	番茄含有抗氧化剂，因此，黄瓜含有抗氧化剂
29	郁金香易得灰霉病，因此，花易得灰霉病	郁金香易得灰霉病，因此，百合易得灰霉病

30	白杨树有22对染色体，因此，树有22对染色体	白杨树有22对染色体，因此，苹果树有22对染色体
31	狗有基因N，因此，仓鼠有基因N	狗有基因N，因此，四足动物有基因N
32	鹰有两个肝脏室，因此，莺有两个肝脏室	鹰有两个肝脏室，因此，鸟有两个肝脏室
33	苍蝇有心脏，因此，马蜂有心脏	苍蝇有心脏，因此，昆虫有心脏
34	鳟鱼有嗅叶，因此，食人鱼有嗅叶	鳟鱼有嗅叶，因此，鱼有嗅叶
35	响尾蛇有一个骨盆，因此，玉米蛇有一个骨盆	响尾蛇有一个骨盆，因此，蛇有一个骨盆
36	橘子有gamma属性，因此，杏有gamma属性	橘子有gamma属性，因此，水果有gamma属性
37	玫瑰花含有羽状复叶，因此，紫罗兰含有羽状复叶	玫瑰花含有羽状复叶，因此，花含有羽状复叶
38	苹果树会产生植物激素，因此，树会产生植物激素	苹果树会产生植物激素，因此，蓝色云杉会产生植物激素
39	狮子有38对染色体，因此，四足动物有38对染色体	狮子有38对染色体，因此，乌龟有38对染色体
40	鹦鹉生存需要镁，因此，鸟生存需要镁	鹦鹉生存需要镁，因此，秃鹫生存需要镁
41	蜜蜂有触角，因此，昆虫有触角	蜜蜂有触角，因此，跳蚤有触角
42	眼镜蛇有心包，因此，蛇有心包	眼镜蛇有心包，因此，棉口蛇有心包
43	葡萄含有蛋白质K，因此，水果含有蛋白质K	葡萄含有蛋白质K，因此，杨桃含有蛋白质K
44	黄瓜含有基因P，因此，蔬菜含有基因P	黄瓜含有基因P，因此，夏南瓜含有基因P
45	百合花由鳞茎繁殖，因此，花由鳞茎繁殖	百合花由鳞茎繁殖，因此，紫丁香由鳞茎繁殖
46	狗有基因N，因此，猫有基因N	狗有基因N，因此，四足动物有基因N
47	鹰有两个肝脏室，因此，乌鸦有两个肝脏室	鹰有两个肝脏室，因此，鸟有两个肝脏室
48	苍蝇有心脏，因此，蚂蚁有心脏	苍蝇有心脏，因此，昆虫有心脏
49	鳟鱼有嗅叶，因此，金鱼有嗅叶	鳟鱼有嗅叶，因此，鱼有嗅叶
50	响尾蛇有一个骨盆，因此，眼镜蛇有一个骨盆	响尾蛇有一个骨盆，因此，蛇有一个骨盆
51	橘子有gamma属性，因此，苹果有gamma属性	橘子有gamma属性，因此，水果有gamma属性
52	玫瑰花含有羽状复叶，因此，郁金香含有羽状复叶	玫瑰花含有羽状复叶，因此，花含有羽状复叶
53	苹果树会产生植物激素，因此，树会产生植物激素	苹果树会产生植物激素，因此，松树会产生植物激素
54	狮子有38对染色体，因此，四足动物有38对染色体	狮子有38对染色体，因此，马有38对染色体
55	鹦鹉生存需要镁，因此，鸟生存需要镁	鹦鹉生存需要镁，因此，乌鸦生存需要镁
56	蜜蜂有触角，因此，昆虫有触角	蜜蜂有触角，因此，蜘蛛有触角
57	眼镜蛇有心包，因此，蛇有心包	眼镜蛇有心包，因此，响尾蛇有心包
58	葡萄含有蛋白质K，因此，水果含有蛋白质K	葡萄含有蛋白质K，因此，香蕉含有蛋白质K
59	黄瓜含有基因P，因此，蔬菜含有基因P	黄瓜含有基因P，因此，西红柿含有基因P
60	百合花由鳞茎繁殖，因此，花由鳞茎繁殖	百合花由鳞茎繁殖，因此，玫瑰花由鳞茎繁殖

Part 2 Explicit tasks

No.	Up	Down
1	所有狗都有基因N，因此，所有仓鼠都有基因N	所有狗都有基因N，因此，所有四足动物都有基因N
2	所有鹰都有两个肝脏室，因此，所有莺都有两个肝脏室	所有鹰都有两个肝脏室，因此，所有鸟都有两个肝脏室
3	所有苍蝇都有心脏，因此，所有马蜂都有心脏	所有苍蝇都有心脏，因此，所有昆虫都有心脏
4	所有鳟鱼都有嗅叶，因此，所有食人鱼都有嗅叶	所有鳟鱼都有嗅叶，因此，所有鱼都有嗅叶
5	所有响尾蛇都有一个骨盆，因此，所有玉米蛇都有一个骨盆	所有响尾蛇都有一个骨盆，因此，所有蛇都有一个骨盆
6	所有橘子都有gamma属性，因此，所有杏都有gamma属性	所有橘子都有gamma属性，因此，所有水果都有gamma属性
7	所有玫瑰花都含有羽状复叶，因此，所有紫罗兰都含有羽状复叶	所有玫瑰花都含有羽状复叶，因此，所有花都含有羽状复叶
8	所有苹果树都会产生植物激素，因此，所有蓝色云杉都会产生植物激素	所有苹果树都会产生植物激素，因此，所有树都会产生植物激素
9	所有狮子都有38对染色体，因此，所有四足动物都有38对染色体	所有狮子都有38对染色体，因此，所有乌龟都有38对染色体
10	所有鹦鹉生存都需要镁，因此，所有鸟生存都需要镁	所有鹦鹉生存都需要镁，因此，所有秃鹫生存都需要镁
11	所有蜜蜂都有触角，因此，所有昆虫都有触角	所有蜜蜂都有触角，因此，所有跳蚤都有触角
12	所有眼镜蛇都有心包，因此，所有蛇都有心包	所有眼镜蛇都有心包，因此，所有棉口蛇都有心包
13	所有葡萄都含有蛋白质K，因此，所有水果都含有蛋白质K	所有葡萄都含有蛋白质K，因此，所有杨桃都含有蛋白质K
14	所有黄瓜都含有基因P，因此，所有蔬菜都含有基因P	所有黄瓜都含有基因P，因此，所有夏南瓜都含有基因P
15	所有百合花都由鳞茎繁殖，因此，所有花都由鳞茎繁殖	所有百合花都由鳞茎繁殖，因此，所有紫丁香都由鳞茎繁殖
16	所有狗都有基因N，因此，所有猫都有基因N	所有狗都有基因N，因此，所有四足动物都有基因N
17	所有鹰都有两个肝脏室，因此，所有乌鸦都有两个肝脏室	所有鹰都有两个肝脏室，因此，所有鸟都有两个肝脏室
18	所有苍蝇都有心脏，因此，所有蚂蚁都有心脏	所有苍蝇都有心脏，因此，所有昆虫都有心脏
19	所有鳟鱼都有嗅叶，因此，所有金鱼都有嗅叶	所有鳟鱼都有嗅叶，因此，所有鱼都有嗅叶
20	所有响尾蛇都有一个骨盆，因此，所有眼镜蛇都有一个骨盆	所有响尾蛇都有一个骨盆，因此，所有蛇都有一个骨盆
21	所有橘子都有gamma属性，因此，所有苹果都有gamma属性	所有橘子都有gamma属性，因此，所有水果都有gamma属性
22	所有玫瑰花都含有羽状复叶，因此，所有郁金香都含有羽状复叶	所有玫瑰花都含有羽状复叶，因此，所有花都含有羽状复叶
23	所有苹果树都会产生植物激素，因此，所有松树都会产生植物激素	所有苹果树都会产生植物激素，因此，所有树都会产生植物激素
24	所有狮子都有38对染色体，因此，所有四足动物都有38对染色体	所有狮子都有38对染色体，因此，所有马都有38对染色体
25	所有鹦鹉生存都需要镁，因此，所有鸟生存都需要镁	所有鹦鹉生存都需要镁，因此，所有乌鸦生存都需要镁
26	所有蜜蜂都有触角，因此，所有昆虫都有触角	所有蜜蜂都有触角，因此，所有蜘蛛都有触角
27	所有眼镜蛇都有心包，因此，所有蛇都有心包	所有眼镜蛇都有心包，因此，所有响尾蛇都有心包
28	所有葡萄都含有蛋白质K，因此，所有水果都含有蛋白质K	所有葡萄都含有蛋白质K，因此，所有香蕉都含有蛋白质K

29	所有黄瓜都含有基因P，因此，所有蔬菜都含有基因P	所有黄瓜都含有基因P，因此，所有西红柿都含有基因P
30	所有百合花都由鳞茎繁殖，因此，所有花都由鳞茎繁殖	所有百合花都由鳞茎繁殖，因此，所有玫瑰花都由鳞茎繁殖
31	所有猫都有T细胞，因此，所有麋鹿都有T细胞	所有猫都有T细胞，因此，所有四足动物都有T细胞
32	所有麻雀都有籽骨，因此，所有火烈鸟都有籽骨	所有麻雀都有籽骨，因此，所有鸟都有籽骨
33	所有蚂蚁都含有内质网，因此，所有蠕虫都含有内质网	所有蚂蚁都含有内质网，因此，所有昆虫都含有内质网
34	所有鲈鱼都有一个鳔，因此，所有甲鱼都有一个鳔	所有鲈鱼都有一个鳔，因此，所有鱼都有一个鳔
35	所有苹果都含有维生素D，因此，所有油桃都含有维生素D	所有苹果都含有维生素D，因此，所有水果都含有维生素D
36	所有胡萝卜都含有铁，因此，所有青椒都含有铁	所有胡萝卜都含有铁，因此，所有蔬菜都含有铁
37	所有松树都在钙质土壤生长良好，因此，所有桃树都在钙质土壤生长良好	所有松树都在钙质土壤生长良好，因此，所有树都在钙质土壤生长良好
38	所有马都有alpha属性，因此，所有蜥蜴都有alpha属性	所有马都有alpha属性，因此，所有四足动物都有alpha属性
39	所有鸽子都有beta属性，因此，所有鸟都有beta属性	所有鸽子都有beta属性，因此，所有模仿鸟都有beta属性
40	所有蜘蛛都有肺脏，因此，所有昆虫都有肺脏	所有蜘蛛都有肺脏，因此，所有螳螂都有肺脏
41	所有金鱼都含有巨噬细胞，因此，所有鱼都含有巨噬细胞	所有金鱼都含有巨噬细胞，因此，所有鲸鱼都含有巨噬细胞
42	所有香蕉都含有钾，因此，所有水果都含有钾	所有香蕉都含有钾，因此，所有甜瓜都含有钾
43	所有番茄都含有抗氧化剂，因此，所有蔬菜都含有抗氧化剂	所有番茄都含有抗氧化剂，因此，所有马铃薯都含有抗氧化剂
44	所有郁金香都易得灰霉病，因此，所有花都易得灰霉病	所有郁金香都易得灰霉病，因此，所有鸢尾花都易得灰霉病
45	所有白杨树都有22对染色体，因此，所有树都有22对染色体	所有白杨树都有22对染色体，因此，所有锡卡莫尔树都有22对染色体
46	所有猫都有T细胞，因此，所有狗都有T细胞	所有猫都有T细胞，因此，所有四足动物都有T细胞
47	所有麻雀都有籽骨，因此，所有鹦鹉都有籽骨	所有麻雀都有籽骨，因此，所有鸟都有籽骨
48	所有蚂蚁都含有内质网，因此，所有苍蝇都含有内质网	所有蚂蚁都含有内质网，因此，所有昆虫都含有内质网
49	所有鲈鱼都有一个鳔，因此，所有金鱼都有一个鳔	所有鲈鱼都有一个鳔，因此，所有鱼都有一个鳔
50	所有苹果都含有维生素D，因此，所有橘子都含有维生素D	所有苹果都含有维生素D，因此，所有水果都含有维生素D
51	所有胡萝卜都含有铁，因此，所有青椒都含有铁	所有胡萝卜都含有铁，因此，所有蔬菜都含有铁
52	所有松树都在钙质土壤生长良好，因此，所有桃树都在钙质土壤生长良好	所有松树都在钙质土壤生长良好，因此，所有树都在钙质土壤生长良好
53	所有马都有alpha属性，因此，所有狮子都有alpha属性	所有马都有alpha属性，因此，所有四足动物都有alpha属性
54	所有鸽子都有beta属性，因此，所有鸟都有beta属性	所有鸽子都有beta属性，因此，所有麻雀都有beta属性
55	所有蜘蛛都有肺脏，因此，所有昆虫都有肺脏	所有蜘蛛都有肺脏，因此，所有蜜蜂都有肺脏
56	所有金鱼都含有巨噬细胞，因此，所有鱼都含有巨噬细胞	所有金鱼都含有巨噬细胞，因此，所有鳟鱼都含有巨噬细胞
57	所有香蕉都含有钾，因此，所有水果都含有钾	所有香蕉都含有钾，因此，所有葡萄都含有钾
58	所有番茄都含有抗氧化剂，因此，所有蔬菜都含有抗氧化剂	所有番茄都含有抗氧化剂，因此，所有黄瓜都含有抗氧化剂

59	所有郁金香都易得灰霉病，因此，所有花都易得灰霉病	所有郁金香都易得灰霉病，因此，所有百合都易得灰霉病
60	所有白杨树都有22对染色体，因此，所有树都有22对染色体	所有白杨树都有22对染色体，因此，所有苹果树都有22对染色体

Experimental stimuli in English

Part 1 Implicit tasks

No.	Up	Down
1	Cats have T cell, thus, elks have T cell	Cats have T cell, thus, four-footed animals have T cell
2	Sparrows have sesamoid bones, thus, flamingos have sesamoid bones	Sparrows have sesamoid bones, thus, birds have sesamoid bones
3	Ants contain retinum, thus, worms contain retinum	Ants contain retinum, thus, insects contain retinum
4	Bass have a swim bladder, thus, snappers have a swim bladder	Bass have a swim bladder, thus, fishes have a swim bladder
5	Apples have vitamin D, thus, nectarines have vitamin D	Apples have vitamin D, thus, fruits have vitamin D
6	Carrots contain iron, thus, green peppers contain iron	Carrots contain iron, thus, vegetables contain iron
7	Pines grow well in calcareous soils, thus, peaches grow well in calcareous soils	Pines grow well in calcareous soils, thus, trees grow well in calcareous soils
8	Horses have the property alpha, thus, four-footed animals have the property alpha	Horses have the property alpha, thus, lizards have the property alpha
9	Pigeons have the property beta, thus, birds have the property beta	Pigeons have the property beta, thus, mockingbirds have the property beta
10	Spiders have lungs, thus, insects have lungs	Spiders have lungs, thus, praying mantis have lungs
11	Goldfishes have macrophages cells, thus, fished have macrophages cells	Goldfishes have macrophages cells, thus, whales have macrophages cells
12	Bananas contain potassium, thus, fruits contain potassium	Bananas contain potassium, thus, honeydew(melon) contain potassium
13	Tomatos contain antioxidants, thus, vegetables contain antioxidants	Tomatos contain antioxidants, thus, potatoes contain antioxidants
14	Tulips are susceptible to the botrytis disease, thus, flowers are susceptible to the botrytis disease	Tulips are susceptible to the botrytis disease, thus, iris are susceptible to the botrytis disease
15	Aspens have 22 chromosomes, thus, trees have 22 chromosomes	Aspens have 22 chromosomes, thus, sycamores have 22 chromosomes
16	Cats have T cell, thus, dogs have T cell	Cats have T cell, thus, four-footed animals have T cell
17	Sparrows have sesamoid bones, thus, parrots have sesamoid bones	Sparrows have sesamoid bones, thus, birds have sesamoid bones
18	Ants contain retinum, thus, flies contain retinum	Ants contain retinum, thus, insects contain retinum
19	Bass have a swim bladder, thus, goldfishes have a swim bladder	Bass have a swim bladder, thus, fishes have a swim bladder
20	Apples have vitamin D, thus, oranges have vitamin D	Apples have vitamin D, thus, fruits have vitamin D
21	Carrots contain iron, thus, green peppers contain iron	Carrots contain iron, thus, vegetables contain iron
22	Pines grow well in calcareous soils, thus, peaches grow well in calcareous soils	Pines grow well in calcareous soils, thus, trees grow well in calcareous soils
23	Horses orses have the property alpha, thus, four-footed animals have the property alpha	Horses have the property alpha, thus, lions have the property alpha
24	Pigeons have the property beta, thus, birds have the property beta	Pigeons have the property beta, thus, sparrows have the property beta
25	Spiders have lungs, thus, insects have lungs	Spiders have lungs, thus, bees mantis have lungs
26	Goldfishes have macrophages cells, thus, fished have macrophages cells	Goldfishes have macrophages cells, thus, trouts have macrophages cells

27	Bananas contain potassium, thus, fruits contain potassium	Bananas contain potassium, thus, grapes contain potassium
28	Tomatos contain antioxidants, thus, vegetables contain antioxidants	Tomatos contain antioxidants, thus, cucumbers contain antioxidants
29	Tulips are susceptible to the botrytis disease, thus, flowers are susceptible to the botrytis disease	Tulips are susceptible to the botrytis disease, thus, lilies are susceptible to the botrytis disease
30	Aspens have 22 chromosomes, thus, trees have 22 chromosomes	Aspens have 22 chromosomes, thus, apples have 22 chromosomes
31	Dogs have N genes,thus,hamsters have N genes	Dogs have N genes, thus, four-footed animals have N genes
32	Eagles have a liver with two chambers,thus,orioles have a liver with two chambers	Eagles have a liver with two chambers, thus, birds have a liver with two chambers
33	Flies have hearts, thus, hornets have hearts	Flies have hearts, thus, insects have hearts
34	Trouts have the olfactory lobes, thus, piranhas have the olfactory lobes	Trouts have the olfactory lobes, thus, fishes have olfactory lobes
35	Rattles retain a pelvic girdle, thus, corns retain a pelvic girdle	Rattles retain a pelvic girdle, thus, snakes retain a pelvic girdle
36	Oranges have the property gamma, thus, apricots have the property gamma	Oranges have the property gamma, thus, fruits have the property gamma
37	Roses have pinnately compound, thus, violets have pinnately compound	Roses have pinnately compound, thus, flowers have pinnately compound
38	Apple trees produce the plant hormone ETN, thus, trees produce the plant hormone ETN	Apple trees produce the plant hormone ETN, thus, blue spruces produce the plant hormone ETN
39	Lions have 38 chromosomes, thus, four-footed animals have 38 chromosomes	Lions have 38 chromosomes, thus, turtles have 38 chromosomes
40	Parrots require magnesium to live, thus, birds require magnesium to live	Parrots require magnesium to live, thus, vultures require magnesium to live
41	Bees have antennae, thus, insects have antennae	Bees have antennae, thus, fleas have antennae
42	Cobras have the pericardium, thus, snakes have the pericardium	Cobras have the pericardium, thus, cottonmouthes have the pericardium
43	Grapes contain the protein K, thus, fruits contain the protein K	Grapes contain the protein K, thus, star fruits contain the protein K
44	Cucumbers have the gene P, thus, vegetables have the gene P	Cucumbers have the gene P, thus, zucchini have the gene P
45	Lilies can be propagated by the bulb, thus, flowers can be propagated by the bulb	Lilies can be propagated by the bulb, thus, lilacs can be propagated by the bulb
46	Dogs have N genes, thus, cats have N genes	Dogs have N genes, thus, four-footed animals have N genes
47	Eagles have a liver with two chambers, thus, crows have a liver with two chambers	Eagles have a liver with two chambers, thus, birds have a liver with two chambers
48	Flies have hearts, thus, ants have hearts	Flies have hearts, thus, insects have hearts
49	Trouts have the olfactory lobes, thus, goldfishes have the olfactory lobes	Trouts have the olfactory lobes, thus, fishes have olfactory lobes
50	Rattles retain a pelvic girdle, thus, cobras retain a pelvic girdle	Rattles retain a pelvic girdle, thus, snakes retain a pelvic girdle
51	Oranges have the property gamma, thus, apples the property gamma	Oranges have the property gamma, thus, fruits have the property gamma
52	Roses have pinnately compound, thus, tulips have pinnately compound	Roses have pinnately compound, thus, flowers have pinnately compound
53	Apple trees produce the plant hormone ETN, thus, trees produce the plant	Apple trees produce the plant hormone ETN, thus, pine trees produce the plant

	hormone ETN	hormone ETN
54	Lions have 38 chromosomes, thus, four-footed animals have 38 chromosomes	Lions have 38 chromosomes, thus, horses have 38 chromosomes
55	Parrots require magnesium to live, thus, birds require magnesium to live	Parrots require magnesium to live, thus, crows require magnesium to live
56	Bees have antennae, thus, insects have antennae	Bees have antennae, thus, spiders have antennae
57	Cobras have the pericardium, thus, snakes have the pericardium	Cobras have the pericardium, thus, rattles have the pericardium
58	Grapes contain the protein K, thus, fruits contain the protein K	Grapes contain the protein K, thus, bananas contain the protein K
59	Cucumbers have the gene P, thus, vegetables have the gene P	Cucumbers have the gene P, thus, tomatoes have the gene P
60	Lilies can be propagated by the bulb, thus, flowers can be propagated by the bulb	Lilies can be propagated by the bulb, thus, roses can be propagated by the bulb

Part 2 Explicit tasks

No.	Up	Down
1	All dogs have N genes, thus, all hamsters have N genes	All dogs have N genes, thus, all four-footed animals have N genes
2	All eagles have a liver with two chambers, thus, all orioles have a liver with two chambers	All eagles have a liver with two chambers, thus, all birds have a liver with two chambers
3	All flies have hearts, thus, all hornets have hearts	All flies have hearts, thus, all insects have hearts
4	All trouts have the olfactory lobes, thus, all piranhas have the olfactory lobes	All trouts have the olfactory lobes, thus, all fishes have olfactory lobes
5	All rattles retain a pelvic girdle, thus, all corns retain a pelvic girdle	All rattles retain a pelvic girdle, thus, all snakes retain a pelvic girdle
6	All oranges have the property gamma, thus, all apricots have the property gamma	All oranges have the property gamma, thus, all fruits have the property gamma
7	All roses have pinnately compound, thus, all violets have pinnately compound	All roses have pinnately compound, thus, all flowers have pinnately compound
8	All apple trees produce the plant hormone ETN, thus, all blue spruces produce the plant hormone ETN	All apple trees produce the plant hormone ETN, thus, all trees produce the plant hormone ETN
9	All lions have 38 chromosomes, thus, all four-footed animals have 38 chromosomes	All lions have 38 chromosomes, thus, all turtles have 38 chromosomes
10	All parrots require magnesium to live, thus, all birds require magnesium to live	All parrots require magnesium to live, thus, all vultures require magnesium to live
11	All bees have antennae, thus, all insects have antennae	All bees have antennae, thus, all fleas have antennae
12	All cobras have the pericardium, thus, all snakes have the pericardium	All cobras have the pericardium, thus, all cottonmouthes have the pericardium
13	All grapes contain the protein K, thus, all fruits contain the protein K	All grapes contain the protein K, thus, all star fruits contain the protein K
14	All cucumbers have the gene P, thus, all vegetables have the gene P	All cucumbers have the gene P, thus, all zucchini have the gene P
15	All lilies can be propagated by the bulb, thus, all flowers can be propagated by	All lilies can be propagated by the bulb, thus, all lilacs can be propagated by the

	the bulb	bulb
16	All dogs have N genes, thus, all cats have N genes	All dogs have N genes, thus, all four-footed animals have N genes
17	All eagles have a liver with two chambers, thus, all crows have a liver with two chambers	All eagles have a liver with two chambers, thus, all birds have a liver with two chambers
18	All flies have hearts, thus, all ants have hearts	All flies have hearts, thus, all insects have hearts
19	All trouts have the olfactory lobes, thus, all goldfishes have the olfactory lobes	All trouts have the olfactory lobes, thus, all fishes have olfactory lobes
20	All rattles retain a pelvic girdle, thus, all cobras retain a pelvic girdle	All rattles retain a pelvic girdle, thus, all snakes retain a pelvic girdle
21	All oranges have the property gamma, thus, all apples the property gamma	All oranges have the property gamma, thus, all fruits have the property gamma
22	All roses have pinnately compound, thus, all lilies have pinnately compound	All roses have pinnately compound, thus, all flowers have pinnately compound
23	All apple trees produce the plant hormone ETN, thus, all pine trees produce the plant hormone ETN	All apple trees produce the plant hormone ETN, thus, all trees produce the plant hormone ETN
24	All lions have 38 chromosomes, thus, all four-footed animals have 38 chromosomes	All lions have 38 chromosomes, thus, all horses have 38 chromosomes
25	All parrots require magnesium to live, thus, all birds require magnesium to live	All parrots require magnesium to live, thus, all crows require magnesium to live
26	All bees have antennae, thus, all insects have antennae	All bees have antennae, thus, all spiders have antennae
27	All cobras have the pericardium, thus, all snakes have the pericardium	All cobras have the pericardium, thus, all rattles have the pericardium
28	All grapes contain the protein K, thus, all fruits contain the protein K	All grapes contain the protein K, thus, all bananas contain the protein K
29	All cucumbers have the gene P, thus, all vegetables have the gene P	All cucumbers have the gene P, thus, all tomatoes have the gene P
30	All lilies can be propagated by the bulb, thus, all flowers can be propagated by the bulb	All lilies can be propagated by the bulb, thus, all roses can be propagated by the bulb
31	All cats have T cell, thus, all elks have T cell	All cats have T cell, thus, all four-footed animals have T cell
32	All sparrows have sesamoid bones, thus, all flamingos have sesamoid bones	All sparrows have sesamoid bones, thus, all birds have sesamoid bones
33	All ants contain retinum, thus, all worms contain retinum	All ants contain retinum, thus, all insects contain retinum
34	All bass have a swim bladder, thus, all snappers have a swim bladder	All bass have a swim bladder, thus, all fishes have a swim bladder
35	All apples have vitamin D, thus, all nectarines have vitamin D	All apples have vitamin D, thus, all fruits have vitamin D
36	All carrots contain iron, thus, all green peppers contain iron	All carrots contain iron, thus, all vegetables contain iron
37	All pines grow well in calcareous soils, thus, all peaches grow well in calcareous soils	All pines grow well in calcareous soils, thus, all trees grow well in calcareous soils
38	All horses have the property alpha, thus, all lizards have the property alpha	All horses have the property alpha, thus, all four-footed animals have the property alpha
39	All pigeons have the property beta, thus, all birds have the property beta	All pigeons have the property beta, thus, all mockingbirds have the property beta
40	All spiders have lungs, thus, all insects have lungs	All spiders have lungs, thus, all praying mantis have lungs

41	All goldfishes have macrophages cells, thus, all fished have macrophages cells	All goldfishes have macrophages cells, thus, all whales have macrophages cells
42	All bananas contain potassium, thus, all fruits contain potassium	All bananas contain potassium, thus, all honeydew(melon) contain potassium
43	All tomatos contain antioxidants, thus, all vegetables contain antioxidants	All tomatos contain antioxidants, thus, all potatoes contain antioxidants
44	All tulips are susceptible to the botrytis disease, thus, all flowers are susceptible to the botrytis disease	All tulips are susceptible to the botrytis disease, thus, all iris are susceptible to the botrytis disease
45	All aspens have 22 chromosomes, thus, all trees have 22 chromosomes	All aspens have 22 chromosomes, thus, all sycamores have 22 chromosomes
46	All cats have T cell, thus, all dogs have T cell	All cats have T cell, thus, all four-footed animals have T cell
47	All sparrows have sesamoid bones, thus, all parrots have sesamoid bones	All sparrows have sesamoid bones, thus, all birds have sesamoid bones
48	All ants contain retinum, thus, all flies contain retinum	All ants contain retinum, thus, all insects contain retinum
49	All bass have a swim bladder, thus, all tunas have a swim bladder	All bass have a swim bladder, thus, all fishes have a swim bladder
50	All apples have vitamin D, thus, all oranges have vitamin D	All apples have vitamin D, thus, all fruits have vitamin D
51	All carrots contain iron, thus, all green peppers contain iron	All carrots contain iron, thus, all vegetables contain iron
52	All pines grow well in calcareous soils, thus, all peaches grow well in calcareous soils	All pines grow well in calcareous soils, thus, all trees grow well in calcareous soils
53	All horses orses have the property alpha, thus, all lions have the property alpha	All horses orses have the property alpha, thus, all four-footed animals have the property alpha
54	All pigeons have the property beta, thus, all birds have the property beta	All pigeons have the property beta, thus, all sparrows have the property beta
55	All spiders have lungs, thus, all insects have lungs	All spiders have lungs, thus, all bees mantis have lungs
56	All goldfishes have macrophages cells, thus, all fished have macrophages cells	All goldfishes have macrophages cells, thus, all trouts have macrophages cells
57	All bananas contain potassium, thus, all fruits contain potassium	All bananas contain potassium, thus, all grapes contain potassium
58	All tomatos contain antioxidants, thus, all vegetables contain antioxidants	All tomatos contain antioxidants, thus, all cucumbers contain antioxidants
59	All tulips are susceptible to the botrytis disease, thus, all flowers are susceptible to the botrytis disease	All tulips are susceptible to the botrytis disease, thus, all lilies are susceptible to the botrytis disease
60	All aspens have 22 chromosomes, thus, all trees have 22 chromosomes	All aspens have 22 chromosomes, thus, all apples have 22 chromosomes

Table S1 Main effect of fallacy and explicitness and the interaction effect of fallacy by explicitness, using reaction times of each trial as covariates. It was found that almost all activations survived (as marked by the blue underlines; the clusters in red means that they were detected when the cluster size threshold was reduced). BA = Brodmann area; MNI = Montreal Neurological Institute; F = fallacy; L = logical; I = implicit; E = explicit;

Brain regions	MNI coordinate			BA	Cluster size	T-score
	x	y	z			
**F-L**						
Rt.Superior Frontal Gyrus	39	42	36	9	39	5.30
Rt.Middle Frontal Gyrus	51	30	36	9		4.49
Rt.Middle Frontal Gyrus	30	45	27	10		3.46
Lt.Insula	-42	15	6	13	92	4.68
Lt.Inferior Frontal Gyrus	-51	18	0	47		4.67
Lt.Superior Parietal Lobule	-33	-66	45	7	137	4.64
Lt.Precuneus	-24	-72	51	7		4.18
Lt.Inferior Parietal Lobule	-42	-57	54	40		3.44
Rt.Superior Frontal Gyrus	3	33	48	8	36	4.62
Rt.Medial Frontal Gyrus	3	39	42	8		4.58
Rt.Superior Parietal Lobule	27	-63	45	7	51	4.17
Rt.Precuneus	15	-72	48	7		3.14
Rt.Inferior Frontal Gyrus	36	24	6	45	27	3.73
	39	15	-15	47		3.32
Lt.Superior Temporal Gyrus	-63	-21	-3	21	28	3.66
Lt.Middle Temporal Gyrus	-51	-30	-3	21		2.94
Lt.Middle Temporal Gyrus	-66	-39	-6	21	6	2.77
**L-F**						
No significant activation						
**E-I**						
No significant activation						
**I-E**						
Rt.Angular Gyrus	33	-63	36	39	27	4.242
Rt.Superior Parietal Lobule	33	-75	45	7		3.623
Rt.Inferior Parietal Lobule	39	-48	54	40	26	4.138

Rt.Superior Parietal Lobule	33	-57	54	7		3.092
Rt.Inferior Parietal Lobule	39	-48	45	40		3.037
**(I_F-I_L)-(E_F-E_L)**						
Rt.Inferior Frontal Gyrus	48	39	15	46	8	3.088
Rt.Precuneus	27	-60	51	7	10	3.066
**(E_F-E_L)-(I_F-I_L)**						
No significant activation						

Table S2 Main effect of fallacy and explicitness and the interaction effect of fallacy by explicitness, using a cutoff of 8 trials in each condition and then 22 subjects were selected. It was found that almost all the activations reported in this manuscript survived (as marked by the blue underlines; the clusters in red means that they were detected when the threshold was reduced) but with lower threshold (uncorrected p <0.005) than the results in the manuscript.

Brain regions	MNI coordinate			BA	Cluster size	T-score
	x	y	z			
**F-L**						
Rt.Medial Frontal Gyrus	6	36	45	8	30	5.37
Lt.Inferior Parietal Lobule	-57	-51	45	40	42	4.06
Lt.Inferior Parietal Lobule	-48	-54	39	40		3.10
Rt.Inferior Parietal Lobule	57	-42	39	40	41	4.02
Rt.Inferior Parietal Lobule	57	-54	42	40		3.37
Lt.Superior Frontal Gyrus	-6	36	51	8	70	3.93
Lt.Superior Frontal Gyrus	-3	21	54	8		3.69
Lt.Medial Frontal Gyrus	-3	45	39	6		3.61
Lt.Inferior Frontal Gyrus	-51	18	12	45	36	3.75
Lt.Insula	-42	12	12	13		3.36
Lt.Middle Temporal Gyrus	-60	-21	-6	21	43	3.53
	-63	-27	-18			3.25
	-45	3	-33		45	3.35
**L-F**						
No significant activation						
**E-I**						
No significant activation						5.91
**I-E**						
Rt.Superior Parietal Lobule	33	-75	45	7	24	3.44
Rt.Precuneus	33	-66	42	19		3.32
**(I_F-I_L)-(E_F-E_L)**						
Rt.Superior Parietal Lobule	24	-69	57	7	7	3.32
**(E_F-E_L)-(I_F-I_L)**						
No significant activation						
